# Radiolabeling of Amberlite IRA-68 Resin Microspheres with Rhenium-188: Optimization and Biodistribution in Tumor-Bearing Mice Following Intratumoral Administration

**DOI:** 10.5812/ijpr-172717

**Published:** 2026-08-03

**Authors:** Zohreh Zavarzadeh-Moghaddam, Mehdi Salehi Barough, Leila Moghaddam-Banaem, Sodeh Sadjadi, Fariba Johari Daha

**Affiliations:** 1 Department of Medical Radiation Engineering, Central Tehran Branch, Islamic Azad University, Tehran, Iran; 2 Nuclear fuel Cycle School, Nuclear Science and Technology Research Institute (NSTRI), Tehran, Iran; 3 Radiation Application Research School, Nuclear Science and Technology Research Institute, Tehran, Iran; 4 Radiation Application School, Nuclear Science and Technology Research Institute (NSTRI), Tehran, Iran

**Keywords:** Rhenium-188, Ion-Exchange Resin, Amberlite IRA-68, Radiopharmaceuticals

## Abstract

**Background:**

Rhenium-188 (^188^Re) is a therapeutic radionuclide with favorable nuclear properties, including high beta energy and a suitable half-life, making it attractive for radiopharmaceutical applications. Solid carriers, such as ion-exchange resins, may improve radionuclide stability, loading efficiency, and controlled delivery.

**Objectives:**

This study aimed to activate Amberlite IRA-68 anion-exchange resin microspheres, optimize their labeling with rhenium and ^188^Re, and evaluate the biodistribution of the radiolabeled microspheres following intratumoral injection in tumor-bearing mice at 24 and 48 h post-administration.

**Methods:**

The effects of contact time, temperature, pH, and initial rhenium concentration on labeling efficiency were systematically investigated. Resin labeling was evaluated by elemental composition analysis using a scanning electron microscope (SEM) equipped with an energy-dispersive X-ray (EDX) analyzer. Radiochemical purity was assessed by instant thin-layer chromatography (ITLC). Activity distribution in mouse organs was assessed using the ISOMED 1010 dose calibrator.

**Results:**

The results demonstrated high labeling efficiency; under optimal conditions (rhenium initial concentration 10 ppm, pH 7, 25 °C, 120 min), rhenium uptake reached approximately 98%. Following intratumoral administration, the radiolabeled microspheres showed high tumor retention (5.39%ID/g at 24 h), whereas radioactivity in the blood and normal organs remained relatively low. This retention indicates localized persistence of the injected microspheres rather than active biological targeting. These findings support the potential of Amberlite IRA-68 resin microspheres as an effective carrier for ^188^Re-based radiopharmaceutical applications.

**Conclusions:**

In this study, Amberlite IRA-68 anion-exchange resin microspheres were successfully evaluated as a carrier system for rhenium and rhenium-188. The results demonstrated high labeling efficiency, reaching approximately 98% under optimized conditions (10 ppm, pH 7, 25 °C, 120 min), indicating a strong affinity between the resin and perrhenate ions. The biodistribution study demonstrated that ^188^Re-labeled Amberlite IRA-68 microspheres showed favorable in vivo characteristics following intratumoral administration. The radiopharmaceutical showed high tumor accumulation at 24 h (%ID/g = 5.39), with persistent retention at 48 h and low blood activity, indicating minimal systemic leakage and limited uptake in critical organs.

## 1. Background

Targeted radionuclide therapy has emerged as a powerful modality in nuclear medicine, enabling the selective delivery of high radiation doses to diseased tissues while minimizing damage to surrounding healthy organs. The success of this approach depends not only on the physical characteristics of the radionuclide but also on the chemical stability, labeling efficiency, and biological behavior of the carrier system used for radionuclide delivery (1, 2).

Among therapeutic radionuclides, ^188^Re has gained considerable attention owing to its favorable nuclear properties. ^188^Re is a high-energy beta emitter (Eβ, max ≈ 2.12 MeV) with a physical half-life of approximately 17 h, making it suitable for the treatment of rapidly proliferating tumors. In addition, ^188^Re emits a 155 keV gamma photon, enabling simultaneous therapeutic application and imaging-based dosimetry (3-6). Its availability from a ^188^W/^188^Re generator system further enhances its clinical appeal by allowing on-site production without the need for a cyclotron (7).

Despite these advantages, the clinical use of ^188^Re requires robust strategies for radionuclide immobilization and delivery. Free rhenium species may exhibit unfavorable biodistribution and rapid clearance, which can reduce therapeutic efficacy and increase radiation exposure to non-target tissues. Therefore, the development of suitable carrier systems capable of providing high labeling efficiency, chemical stability, and controlled radionuclide retention remains a critical challenge in radiopharmaceutical design (4, 6).

Solid carriers, such as microspheres, nanoparticles, and ion-exchange resins, have been extensively investigated for radionuclide immobilization. Among these materials, ion-exchange resins offer several advantages, including high exchange capacity, chemical and mechanical stability, ease of preparation, and strong electrostatic interactions with ionic radionuclide species. These properties make them particularly attractive for applications such as radioembolization and localized radionuclide therapy (8, 9).

Amberlite IRA-68 is a weakly basic anion-exchange resin with a polymeric organic structure containing functional groups that can enable electrostatic binding to negatively charged ions. Its spherical morphology and relatively uniform particle size distribution facilitate efficient solid–liquid contact and favorable ion-exchange kinetics. Previous studies have demonstrated the potential of anion-exchange resins for the uptake of rhenium and technetium analogues, indicating their suitability as carriers for therapeutic radiometals (10-13).

The ion-exchange behavior of rhenium on resins is influenced by several experimental parameters, including contact time, temperature, solution pH, and initial metal concentration. Understanding the effects of these parameters is essential for optimizing labeling conditions and ensuring reproducible, high-efficiency radionuclide loading. Furthermore, detailed surface characterization using scanning electron microscopy (SEM) and energy-dispersive X-ray spectroscopy (EDX) is required to confirm successful rhenium uptake and to evaluate morphological changes after labeling (9, 13).

## 2. Objectives

Several microsphere systems based on ^90^Y, ^166^Ho, and ^188^Re have been investigated for localized radionuclide therapy. However, many of these systems require relatively complex synthesis procedures, expensive materials, or sophisticated radiolabeling protocols. Amberlite IRA-68 is a commercially available, weakly basic anion-exchange resin with high ion-exchange capacity, excellent chemical stability, and simple preparation. To our knowledge, the use of Amberlite IRA-68 as a carrier for ^188^Re has not been systematically investigated. Therefore, the present work aimed to optimize the radiolabeling conditions, characterize the resulting microspheres, and conduct a preliminary evaluation of their in vivo biodistribution following intratumoral administration.

In this study, the procedure was divided into three steps. In the first step, Amberlite IRA-68 resin microspheres were investigated as a carrier for natural rhenium labeling. The effects of contact time, temperature, pH, and initial rhenium concentration on labeling efficiency were systematically evaluated. Surface morphology and elemental composition were characterized using SEM and EDX analyses to confirm successful rhenium immobilization. In the second step, using the data from the first step, microspheres were labeled with rhenium-188 from a ^188^W/^188^Re generator, and radiopharmaceutical quality control was performed. In the last step, biodistribution was investigated in tumor-bearing mice.

## 3. Methods

### 3.1. Materials and Apparatus

Amberlite IRA-68 anion-exchange resin was used as the rhenium carrier. Amberlite IRA-68 resin has a particle size of 20 - 50 mesh, corresponding to approximately 300 - 850 μm. This relatively large particle size favors retention at the injection site after intratumoral administration while minimizing physical migration of intact microspheres.

All chemical reagents, including rhenium metal, hydrogen peroxide solution (30%, H_2_O_2_), sodium hydroxide (NaOH), and hydrochloric acid (HCl), were of analytical grade and were used without further purification.

Scanning electron microscopy (SEM) images and elemental maps were recorded using a ZEISS EVO 18 analytic microscope (Germany) equipped with an energy-dispersive X-ray (EDX) analyzer. Quantitative determination of rhenium uptake was performed by inductively coupled plasma atomic emission spectroscopy (ICP-AES, Optima 7300 DV, USA).

The Tungsten-188/Rhenium-188 generator from PARS-Isotope Company of Iran was used as the source of rhenium-188. A saline solution (0.9% NaCl) was used to extract ^188^ReO_4_^-^ from the alumina-based ^188^Re generator. A dose calibrator (Isomed, Germany) was used to measure the activity of ^188^ReO_4_^-^, which was 500 - 600 mCi. Radiochromatography analysis was conducted using silica gel ITLC chromatography paper from Agilent Technologies US.

Tumor-bearing male mice (8 ± 1 weeks old) were used for the biodistribution studies. The B16-F10 tumor cell line was used, the average tumor volume at the time of injection was 0.5 ± 0.1 mm^2^, injected activity was 100 µCi, injection volume was 100 µL, and the number of animals per group n = 3 was included to ensure reproducibility. The radiolabeled microspheres were administered by direct intratumoral injection.

Activity distribution in mouse organs was evaluated using a dose calibrator ISOMED 1010 (Dresden, Germany). Outcomes are presented as mean ± SD, and statistical evaluation was performed using Student’s t-test, with P values < 0.05 regarded as statistically significant. Male mice, with an average age of 8 ± 1 weeks, were obtained from the animal facility of the Nuclear Science and Research Institute (NSTRI). Animal studies adhered to the regulations established by the United Kingdom Biological Council. The preparation techniques and quality assurance assessments used in this research followed the IAEA guidelines for radiopharmaceutical manufacturing.

Animal studies adhered to the instructions of the United Kingdom Biological Council. Preparation and quality-assurance procedures followed International Atomic Energy Agency guidelines for radiopharmaceutical manufacturing.

### 3.2. Resin Pretreatment

Separate resin samples were treated independently with NaOH and HCl. Each sample was immersed in the respective 50% (w/w) solution at room temperature (25 °C) for 24 h to activate the ion-exchange sites. The suspension was then centrifuged, and the precipitate was dried.

### 3.3. Preparation of Rhenium Solution

Natural rhenium metal was reacted with hydrogen peroxide to form perrhenic acid. Hydrogen peroxide solution (30%) was added to rhenium (400 µL per 8 mg rhenium), and the mixture was stirred in a water bath at 40 °C for 20 min until complete dissolution was achieved.

### 3.4. Labeling Experiments

Labeling experiments were conducted by mixing 5 mL of rhenium solution at concentrations ranging from 10 to 100 ppm with 100 mg of the activated resin. The mixtures were agitated at 200 rpm for contact times of 0.5, 1, 2, 2.5, and 3 h at pH values between 3 and 8. Additional experiments were conducted at different temperatures (25, 35, 45, 55, and 65 °C).

After labeling, the suspensions were filtered, and the equilibrium rhenium concentration was measured using ICP-AES.

Labeling efficiency (R%) was calculated as follows:

$$R\%=\frac{\left(C_{0}-C_{e}\right)100}{C_{0}}$$

Where C_0_ and C_e_ are the initial and equilibrium concentrations (ppm) of rhenium ion, respectively.

### 3.5. Characterization Techniques

The surface morphology of the resin before and after labeling was examined using SEM. Elemental composition and confirmation of rhenium uptake were obtained using EDX analysis.

### 3.6. Radiolabeling of Amberlite IRA-68 With Rhenium-188

After optimizing the labeling conditions with rhenium, radiolabeling with rhenium-188 from the ^188^W/^188^Re generator was carried out in the second stage. In this procedure, a saline solution (0.9% NaCl) was used to extract ^188^ReO_4_^-^ from the alumina-based ^188^Re generator.

Radiochemical purity was determined using ITLC-SG strips as the stationary phase and acetone as the mobile phase. Under these conditions, free ^188^ReO_4_^-^ migrated with the solvent front (Rf≈1), whereas the radiolabeled Amberlite IRA-68 microspheres remained at the origin (Rf≈0). Radiochemical purity was calculated from the distribution of radioactivity along the strip using a radio-TLC scanner.

### 3.7. In Vitro Stability Studies

The in vitro stability of the ^188^Re-Amberlite IRA-68 was evaluated by incubating the complex at room temperature for 48 h after preparation. Radiochemical purity of the complex was determined at regular time intervals using paper chromatography and standard quality control techniques.

### 3.8. Biodistribution Studies in Mice

Biodistribution studies were conducted in mice using ^188^Re-Amberlite IRA-68. The ^188^Re-Amberlite IRA-68 was administered directly into the tumor, and the animals were euthanized at specified time intervals. Organs such as bones, kidneys, liver, heart, lungs, and tumors were collected and assessed for radioactivity using an ISOMED 1010 dose calibrator. The proportion of the administered dose per gram of tissue (%ID/g) was determined for each organ.

## 4. Results

### 4.1. Effect of Contact Time

**Figure 1.**
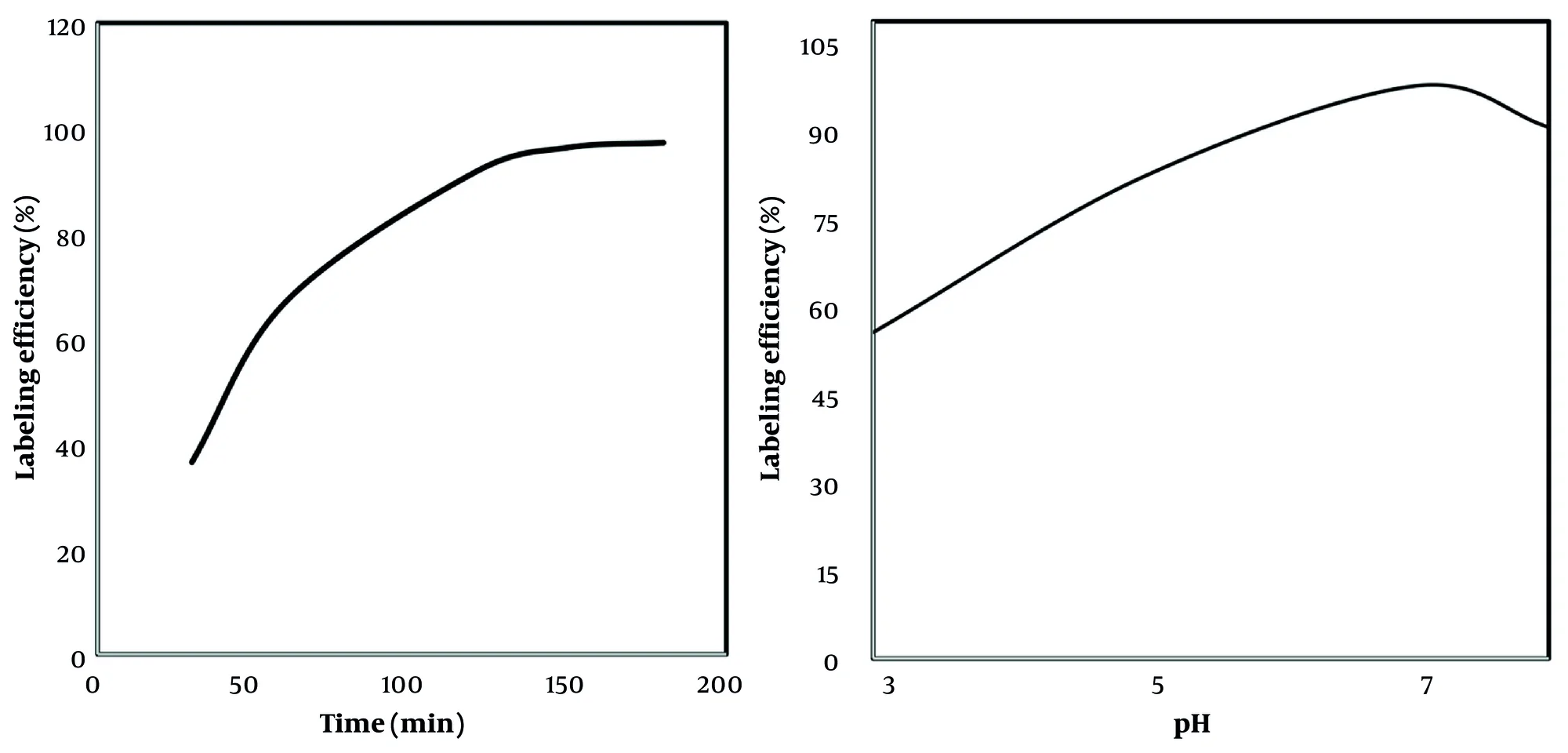
A, Effect of contact time on labeling efficiency of rhenium using Amberlite IRA-68 resin microspheres. B, Effect of pH on labeling efficiency of rhenium using Amberlite IRA-68 resin microspheres.

Figure 1 illustrates the impact of contact time on rhenium uptake under the following experimental conditions: Resin dosage of 100 mg, rhenium concentration of 100 ppm, pH 7, and a temperature of 25 °C. Rhenium ion uptake increased from 36.5% at 30 min to 67.07% at 60 min and reached 91.6% after 120 min. Beyond this time, rhenium ion uptake approached equilibrium, with only marginal increases in labeling efficiency, indicating saturation of active exchange sites and a reduced mass-transfer driving force.

### 4.2. Effect of pH

The effect of pH on the labeling efficiency of rhenium on the Amberlite IRA-68 resin ion exchanger was determined in experiments using 100 mg of resin and 10 mL of 10 ppm rhenium solution (Figure 1B). After shaking for 120 min at 25 °C, the suspension was separated, and the filtrate was analyzed for rhenium ions. The results reveal that labeling efficiency continuously improved with increasing pH, and the maximum labeling efficiency was obtained at pH 7. At low pH levels, protonation of functional groups on the anion-exchange resin decreases the availability of exchange sites. At pH values above the optimum, increased competition for these sites between rhenium ions and hydroxide ions leads to a reduction in labeling efficiency.

### 4.3. Effect of Temperature

The influence of solution temperature on rhenium labeling using Amberlite IRA-68 resin was examined in the temperature range of 25 - 65 °C (labeling conditions: resin dosage 100 mg, rhenium concentration 100 ppm, pH 7, time 120 min). As shown in Table 1, increasing the temperature led to a higher degree of rhenium labeling, although this beneficial effect was not significant. This enhancement in rhenium labeling can be attributed to the increased diffusion rate of rhenium from the solution to the resin surface at higher temperatures. Considering the slight improvement in labeling efficiency with increasing temperature, 25 °C was chosen for subsequent experiments.

**Table 1.** Effect of Temperature on the Labeling Efficiency of Rhenium Using Amberlite IRA-68 Resin Microspheres

Temperature (°C)	Labeling Efficiency (%)
**25**	91.6
**35**	93.22
**45**	94.17
**55**	94.64
**65**	94.96

### 4.4. Effect of Initial Rhenium Concentration

**Figure 2.**
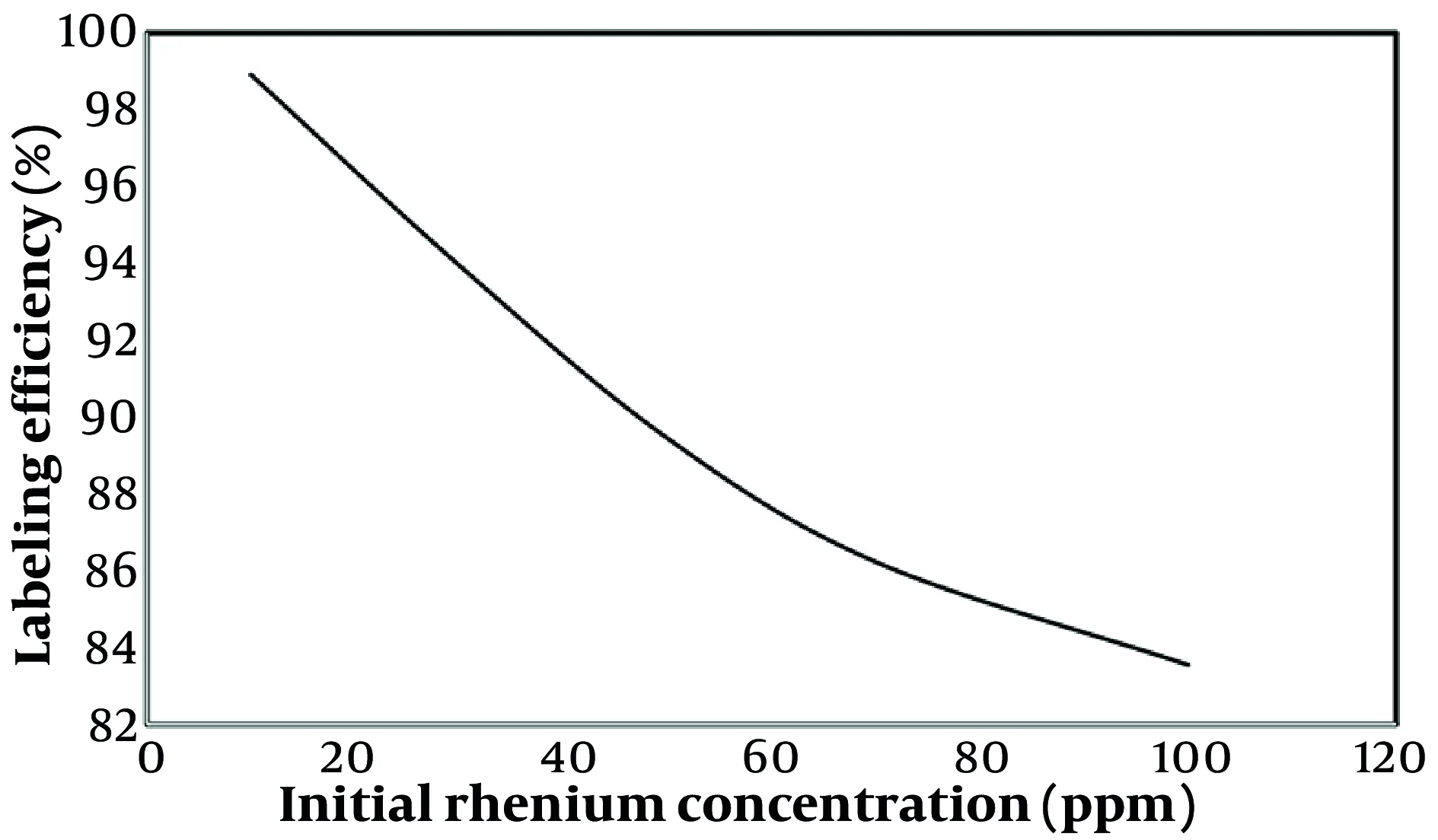
Effect of initial rhenium concentration on labeling efficiency of rhenium using Amberlite IRA-68 resin microspheres.

The effect of different initial rhenium concentrations of 10, 30, 50, 70, 100, and 200 ppm on the labeling efficiency of Amberlite IRA-68 resin was investigated. As depicted in Figure 2, when the initial rhenium concentration was increased from 10 to 100 ppm, labeling efficiency decreased, which may be related to active-site occupancy. The results indicate that the maximum rhenium labelling, 98.8%, can be achieved at an initial concentration of 10 ppm at 25 °C for 120 min at pH 7.

### 4.5. Labeling of Amberlite IRA-68 With Rhenium-188

**Figure 3.**
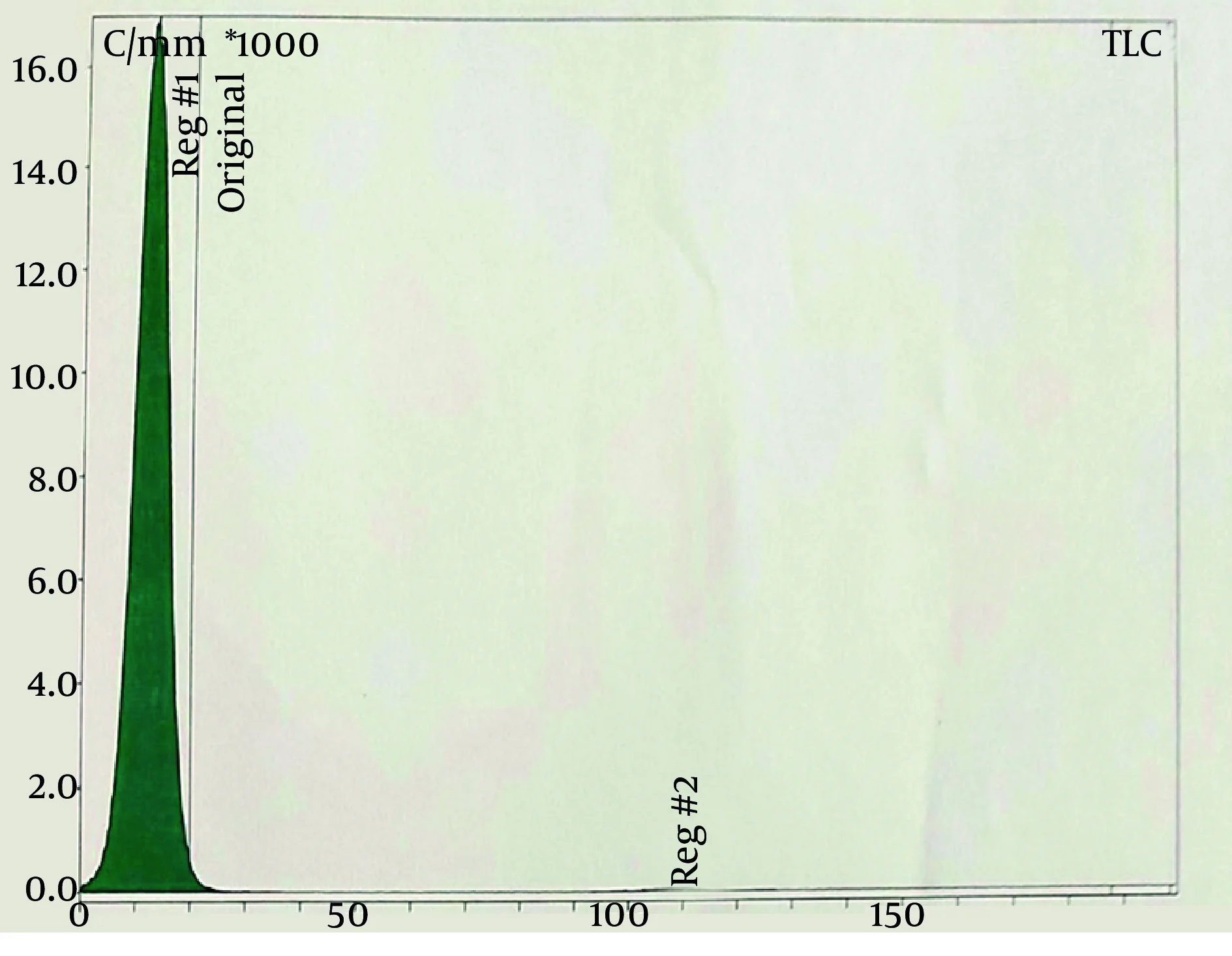
ITLC of ^188^Re- Amberlite IRA-68 complex by Acetone as mobile phase

^188^Re was mixed with 100 mg of the activated resin. The mixture was agitated at 200 rpm for a contact time of 120 min at pH 7 and 25 °C. The suspension was then filtered. The labeling proficiency measured by ITLC is shown in Figure 3, and the result was 98%.

### 4.6. SEM and EDX Analysis

**Figure 4.**
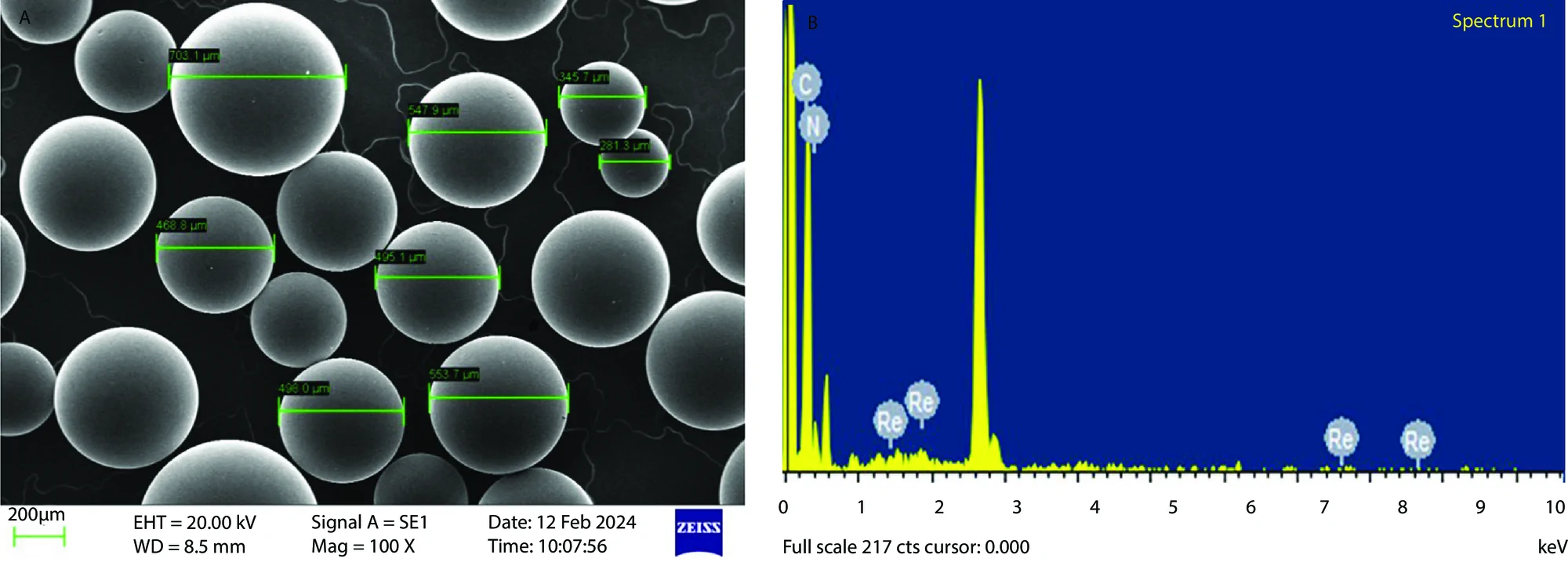
A, SEM; and B, EDX images of Re-resin microsphere.

SEM images revealed that the microspheres maintained their spherical shape and smooth surface morphology after the labeling process. No significant surface degradation, cracking, or aggregation was observed, indicating that the labeling conditions did not adversely affect the physical integrity of the microspheres (Figure 4A). The EDX spectrum (Figure 4B) confirmed the presence of rhenium as an elemental constituent in the Amberlite IRA-68 resin.

### 4.7. In Vitro Stability Studies

**Figure 5.**
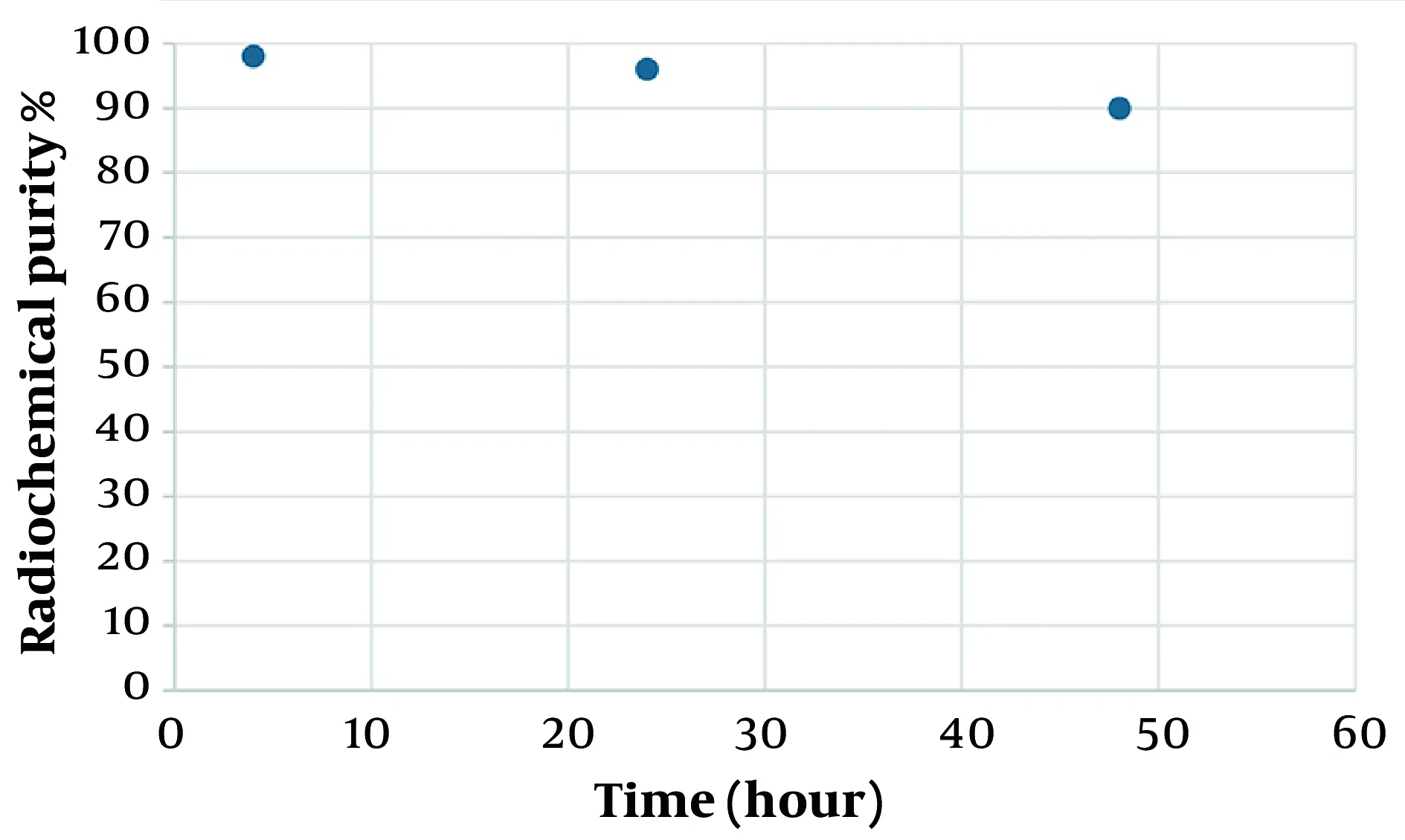
Stability of the ^188^Re- Amberlite IRA-68 complex up to 48 h (pH 7)

The complex exhibited excellent in vitro stability at pH ~ 7 when stored at room temperature. The radiochemical purity under the above-mentioned conditions was found to be retained to an extent of ~ 90% at 48 h post-preparation (Figure 5).

### 4.8. Biodistribution in Mice

**Figure 6.**
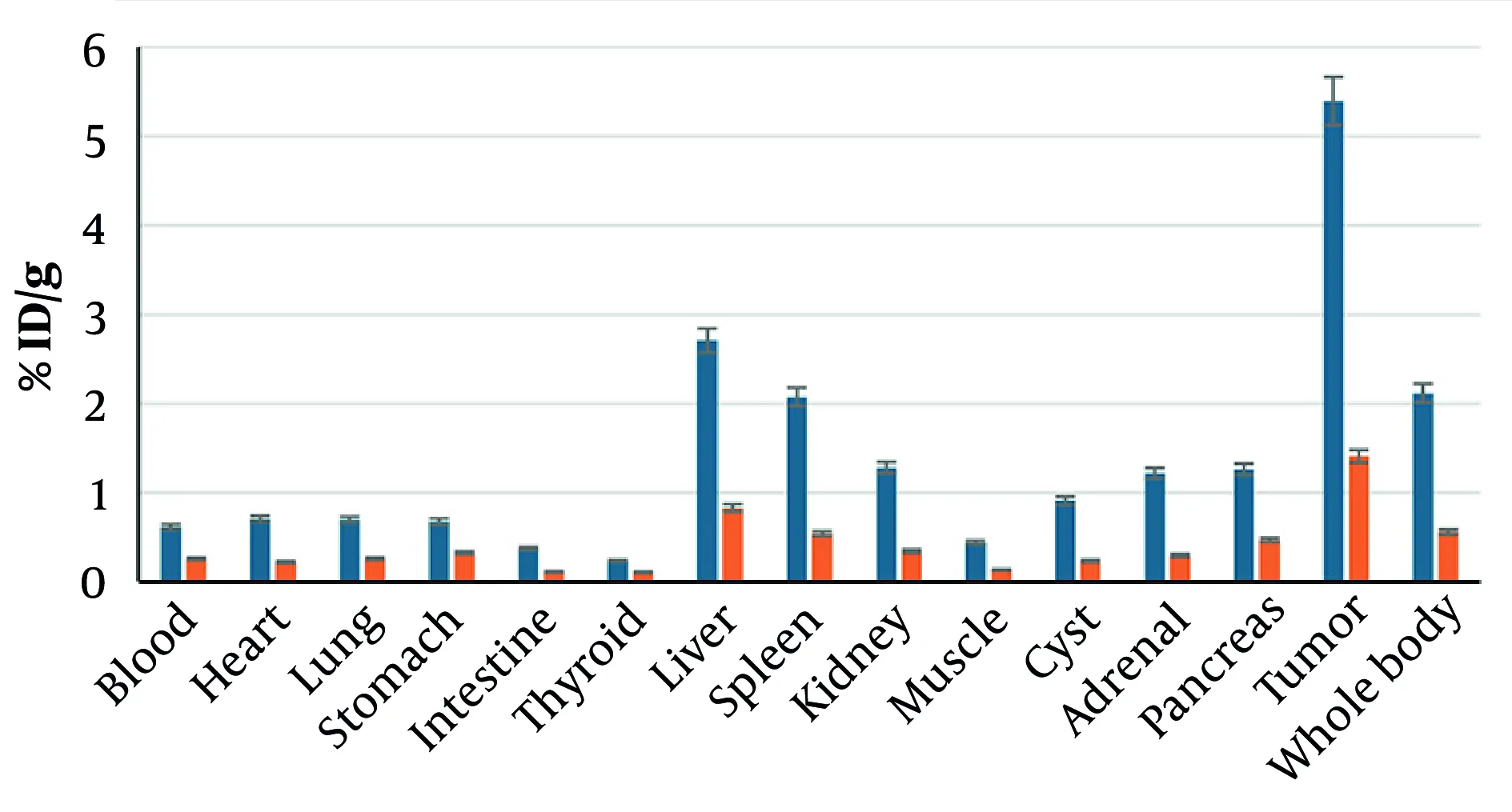
Biodistribution of ^188^Re-labeled Amberlite IRA-68 microspheres following intratumoral injection in tumor-bearing mice at 24 h and 48 h post-injection.(n=3)

The biodistribution of ^188^Re-labeled Amberlite IRA-68 microspheres was evaluated in tumor-bearing mice at 24 h and 48 h post-injection. The percentage of injected dose per gram of tissue (%ID/g) for major organs is shown in Figure 6.

The biodistribution study demonstrated that ^188^Re-IRA-68 microspheres accumulated preferentially in tumor tissue compared with most normal organs, suggesting effective localization after administration. The highest uptake was observed in the tumor, reaching 5.39%ID/g at 24 h, and remained higher than that in all other organs even after decreasing to 1.41%ID/g at 48 h. This prolonged retention is advantageous for radionuclide therapy because it allows sustained delivery of β-particle radiation to the tumor while reducing exposure to surrounding healthy tissues.

One of the most important indicators of targeting efficiency is the tumor-to-blood (T/B) ratio, which reflects the selectivity of the radiopharmaceutical. Based on the present data, the tumor-to-blood ratio was approximately 8.8 at 24 h and 5.5 at 48 h. The tumor-to-organ ratios at both time points are presented in Table 2.

**Table 2.** Tumor-to-Organ Ratios

Ratio	24 h	48 h
**Tumor/Blood**	8.75	5.47
**Tumor/Liver**	1.99	1.70
**Tumor/Kidney**	4.21	4.09
**Tumor/Lung**	7.75	5.42
**Tumor/Muscle**	12.18	10.52

A tumor-to-blood ratio substantially greater than one indicates that the activity concentration in the tumor is much higher than that in circulating blood, minimizing irradiation of the bone marrow and other blood-rich organs. The ratio of nearly nine at 24 h suggests excellent tumor selectivity following clearance from the circulation. Although the ratio decreased at 48 h due to washout of tumor activity, it remained above five, indicating continued preferential retention within the tumor.

The liver exhibited the second-highest uptake (2.71%ID/g at 24 h and 0.83%ID/g at 48 h), which may be attributed to uptake by the reticuloendothelial system or hepatic clearance of microspheres. Similarly, moderate accumulation in the spleen and kidneys reflects physiological filtration and clearance mechanisms. Importantly, uptake in blood, heart, lung, muscle, and thyroid remained low and decreased markedly over time, demonstrating effective systemic clearance and limited nonspecific distribution.

The low thyroid uptake is particularly noteworthy because free perrhenate (^188^ReO_4_^-^) is known to accumulate in the thyroid through the sodium-iodide symporter. The observed thyroid uptake of only 0.24%ID/g at 24 h and 0.11%ID/g at 48 h suggests that the radiolabel remained largely stable in vivo, with minimal release of free ^188^Re.

The favorable tumor localization combined with rapid blood clearance suggests that ^188^Re-IRA-68 microspheres possess pharmacokinetic characteristics desirable for radionuclide therapy. High tumor uptake and high tumor-to-blood ratios increase the likelihood of delivering therapeutic radiation doses to malignant tissue while minimizing radiation exposure to normal organs. Nevertheless, additional dosimetric studies and therapeutic efficacy experiments are required to determine whether the retained activity is sufficient to achieve tumor control while maintaining acceptable normal-organ doses.

These ratios further demonstrate the preferential accumulation of ^188^Re-IRA-68 microspheres in tumor tissue relative to normal organs and indicate promising preclinical characteristics that warrant further evaluation.

## 5. Discussion

Following intratumoral administration, the highest radioactivity was observed within the injected tumor, reflecting retention of the radiolabeled microspheres at the injection site rather than active tumor targeting. Because Amberlite IRA-68 microspheres have diameters of approximately 300 - 850 μm, migration of intact particles from the tumor is expected to be minimal. Therefore, the radioactivity detected in the liver, spleen, and other organs is more likely attributable to the limited release of free ^188^Re-perrhenate or leakage of soluble radioactive species rather than transport of intact microspheres.

The temperature effects suggest that the adsorption process is not strongly endothermic. The observed decrease in efficiency at moderate temperatures may be attributable to partial weakening of electrostatic interactions between the resin functional groups and perrhenate ions. However, the relatively stable adsorption performance at higher temperatures indicates that ion-exchange interactions remain dominant. This thermal stability is advantageous for biomedical applications, in which temperature variations may occur during preparation or administration.

pH critically influences labeling efficiency, as it directly affects the ionization state of the resin functional groups. At low pH values, protonation of amine groups reduces the availability of negatively charged binding sites, leading to lower labeling efficiency. As the pH approaches neutral conditions, deprotonation enhances the electrostatic attraction between the resin and perrhenate ions, resulting in maximum adsorption efficiency. At higher pH values, competition with hydroxide ions becomes significant, reducing binding efficiency. These findings highlight the importance of maintaining an optimal pH environment for effective radiolabeling.

The effect of initial rhenium concentration demonstrates typical saturation behavior. At low concentrations, the high availability of active sites enables nearly complete rhenium uptake, as reflected by the ~98% labeling efficiency. As the concentration increases, the finite number of active sites becomes a limiting factor, leading to reduced labeling efficiency.

Morphological analysis using SEM confirmed that the resin maintains its spherical shape and structural integrity after labeling, which is essential for maintaining consistent flow properties and mechanical stability in applications such as radioembolization. EDX analysis provided direct evidence of successful rhenium incorporation by confirming the presence of rhenium on the resin surface.

Radiolabeling experiments with ^188^Re demonstrated a high radiochemical purity of approximately 98%, indicating efficient binding of the radionuclide and minimal free perrhenate. This is a critical parameter for clinical applications, as free radionuclide can lead to undesirable biodistribution and increased radiation dose to non-target tissues. The consistency between non-radioactive adsorption results and radiolabeling outcomes further supports the reliability of the optimized conditions.

Overall, the labeling mechanism is predominantly governed by electrostatic ion-exchange interactions, supported by favorable kinetics, high efficiency, and structural stability. These characteristics make Amberlite IRA-68 a strong candidate for further development in radiopharmaceutical applications.

The present biodistribution study demonstrates that ^188^Re-labeled Amberlite IRA-68 microspheres exhibit favorable tumor retention following intratumoral administration, with the tumor representing the primary site of radioactivity accumulation throughout the observation period.

The high tumor retention observed at 24 h (5.39%ID/g) reflects efficient deposition of the microspheres within the tumor tissue. Although tumor activity decreased by 48 h, substantial retention (1.41%ID/g) remained, suggesting that the microspheres remained localized sufficiently long to deliver a therapeutic radiation dose. Such prolonged retention is desirable for β-emitting radionuclides such as ^188^Re, whose physical half-life (16.9 h) allows continuous irradiation of tumor cells over several decay cycles.

The relatively low blood activity at both time points indicates minimal leakage of microspheres into the systemic circulation following intratumoral injection. This observation is particularly important because systemic redistribution could increase radiation exposure to healthy organs.

The liver and spleen showed the highest uptake among normal organs, consistent with the physiological role of the reticuloendothelial system (RES) in trapping particulate materials and microspheres. Uptake by Kupffer cells in the liver and macrophages in the spleen is commonly observed for radiolabeled microsphere formulations and suggests partial phagocytic clearance of particles escaping the tumor site.

Although prolonged tumor retention is encouraging for localized radionuclide therapy, therapeutic efficacy depends not only on total retained activity but also on its spatial distribution within the tumor. Because ^188^Re emits high-energy β-particles (max β energy ~2.12 MeV, mean range in tissue ~3 - 4 mm, max ~10 - 11 mm), heterogeneous intratumoral distribution may produce regions receiving suboptimal absorbed doses. Future investigations should therefore include MIRD-based absorbed dose calculations, voxel dosimetry, and imaging studies to evaluate dose homogeneity and estimate therapeutic effectiveness.

The principal strengths of this work include the high radiolabeling efficiency (~98%), a simple preparation procedure, and the preliminary demonstration of in vivo retention following intratumoral administration. Nevertheless, several limitations should be acknowledged. Radiochemical stability under physiological conditions (PBS or human serum albumin), therapeutic efficacy, long-term toxicity, and quantitative dosimetry were not investigated. These aspects will be addressed in future studies.

### 5.1. Conclusions

In this study, Amberlite IRA-68 anion-exchange resin microspheres were successfully evaluated as a carrier system for rhenium and rhenium-188. The results demonstrated high labeling efficiency, reaching approximately 98% under optimized conditions (10 ppm, pH 7, 25 °C, 120 min), indicating strong affinity between the resin and perrhenate ions.

The systematic investigation of key parameters, including contact time, temperature, pH, and initial concentration, enabled optimization of labeling conditions. Successful radiolabeling with ^188^Re, accompanied by high radiochemical purity, confirms the practical applicability of the system for radiopharmaceutical use.

The biodistribution study demonstrated that ^188^Re-labeled Amberlite IRA-68 microspheres possess favorable in vivo characteristics following intratumoral administration. The radiopharmaceutical showed the following:

High tumor accumulation at 24 h (%ID/g = 5.39) with persistent retention at 48 h;

Low blood activity, indicating minimal systemic leakage;

Limited uptake in critical organs such as the brain and thyroid, suggesting good in vivo stability and low nonspecific distribution;

Expected liver and spleen accumulation, consistent with reticuloendothelial clearance of microspheres;

High tumor-to-background ratios, supporting selective localization within tumor tissue.

The present study demonstrates that Amberlite IRA-68 resin microspheres can be efficiently radiolabeled with ^188^Re, achieving high radiochemical purity and favorable retention following intratumoral administration in tumor-bearing mice. The observed biodistribution supports their potential as a localized radionuclide delivery system. However, additional investigations, including radiochemical stability under physiological conditions, therapeutic efficacy studies, SPECT imaging, long-term toxicity assessment, and MIRD-based dosimetry, are required before clinical translation can be considered.

## Data Availability

The dataset presented in the study is available on request from the corresponding author during submission or after publication. The data are not publicly available
